# Redescription of *Urartucoris ermolenkoi* (Hemiptera, Heteroptera, Coreidae) and a revised key to the genera of Pseudophloeini of the Western Palaearctic Region

**DOI:** 10.3897/zookeys.319.4309

**Published:** 2013-07-30

**Authors:** Petr Kment, Meral Fent, George Japoshvili

**Affiliations:** 1 Department of Entomology, National Museum, 14800 Prague 4, Czech Republic; 2 Department of Biology, Faculty of Science, Trakya University, 22030 Edirne, Turkey; 3 Entomology and Biocontrol Research Centre, Agricultural University of Georgia, Tbilisi, Georgia

**Keywords:** Heteroptera, Coreidae, Pseudophloeinae, key, morphology, ecology, distribution, Turkey, Palaearctic Region

## Abstract

*Urartucoris ermolenkoi* P. V. Putshkov, 1979 (Hemiptera: Heteroptera: Coreidae: Pseudophloeinae) is recorded from Turkey for the first time. Redescriptions of the genus and species are provided, the male of *Urartucoris ermolenkoi* being described for the first time, and intraspecific variability of the species is discussed. Adults of *Urartucoris ermolenkoi* were collected from mid April to end of July and in mid September by means of pitfall traps. First data on the habitat of the species are provided: it is epigeic, inhabiting sparse forests and shrublands at higher elevations (ca. 1400–1600 m a.s.l.) in arid regions of central Anatolia. A revised key to the genera of the West Palaearctic Pseudophloeini is provided. Translations of the original descriptions from Russian are given in Appendix.

## Introduction

The subfamily Pseudophloeinae Stål, 1868 is represented in the Palaearctic Region (*sensu* Aukema and Rieger 2006) by two tribes, the mostly tropical Clavigrallini Stål, 1873 with 3 genera and 9 species distributed in the southern border areas – Arabian Peninsula, Iran, Afghanistan, central and southern China, Taiwan, and Japan, and Pseudophloeini with 17 genera and 42 species distributed throughout the region (Dolling 2006, Aukema et al. 2012). Moulet (1995) monographed the Coreoidea and Pyrrhocoroidea fauna of the Euro-Mediterranean region, keying 10 genera and 26 species of Pseudophloeini: *Anoplocerus* Kiritshenko, 1926 (3 species), *Arenocoris* Hahn, 1834 (4 species), *Bathysolen* Fieber, 1860 (2 species), *Bothrostethus* Fieber, 1860 (1 species), *Ceraleptus* A. Costa, 1847 (4 species), *Loxocnemis* Fieber, 1860 (1 species), *Coriomeris* Westwood, 1842 (9 species), *Nemocoris* R. F. Sahlberg, 1848 (1 species), *Strobilotoma* Fieber, 1860 (1 species), and *Ulmicola* Kirkaldy, 1909 (1 species). However, some Eastern-Mediterranean genera and species were omitted from this work, as follows:

- The genus *Microtelocerus* Reuter, 1900 contains two species, *Microtelocerus testaceus* Reuter, 1900 (described from Tajikistan) and *Microtelocerus linnavuorii* Dolling, 1979 (described from Sinai, Egypt). Dolling (2006) listed *Microtelocerus linnavuorii* also from Libya (as a new record) and *Microtelocerus testaceus* also from Iran without further details (no record was listed by Hoberlandt (1989) or Ghahari et al. (2012)). Records of *Microtelocerus testaceus* from Sinai (Egypt) by Priesner and Wagner (1961) and El Hamouly et al. (2010) almost certainly pertain to *Microtelocerus linnavuorii*.
- The monotypic genus *Urartucoris* P. V. Putshkov, 1979 contains *Urartucoris ermolenkoi* P. V. Putshkov, 1979 described based on two females from the Nakhchivan Autonomous Republic of Azerbaijan (Putshkov 1979).
- Moulet (1995) keyed 4 species of *Ceraleptus* and did not deal with *Ceraleptus sartus* Kiritshenko, 1912 which is a Central Asian species also recorded from the Asian part of Turkey (Wagner 1959: 106).
- Moulet (1995) also omitted three species of *Coriomeris* inhabiting Transcaucasia and the Near East: *Ceraleptus armeniacus* Tshernova, 1978 (Armenia, Asian Turkey, Azerbaijan, Iran), *Ceraleptus pallidus* Reuter, 1900 (South European Territory of Russia, Asian Turkey, Lebanon, Syria, Iran, Central Asia up to North-Western China and Afghanistan), and *Ceraleptus validicornis* Jakovlev, 1904 (Armenia, Asian Turkey, Azerbaijan, Georgia, Iran) (Tshernova 1978, Dolling 2006).

The knowledge on the Coreidae fauna of Turkey was recently improved by several faunistic surveys, especially those by Dursun and Fent (2009), Yıldırım et al. (2011), and Dursun (2011), including references to further minor studies. Finally, the Turkish fauna was summarized in form of a checklist by Dursun (2011), listing 17 genera and 31 species, of which 8 genera and 24 species belong to Pseudophloeinae. An additional species, *Gonocerus patellatus* Kiritshenko, 1916 (Coreinae), was recorded from Anatolia by Yıldırım et al. (2011).

Specimens of an additional genus and species so far unrecorded from Turkey, *Urartucoris ermolenkoi*, were recently obtained by the third author. This species has never been recorded after its original description, and its male sex has remained unknown so far. In this contribution we redescribe the genus and species, provide the first description and illustrations of the male of *Urartucoris ermolenkoi*, and give the first information on its habitat preference. An updated key to the West Palaearctic genera of Pseudophloeini is presented as well.

The examined material of *Urartucoris ermolenkoi* was collected during the ongoing systematic studies on insect diversity of the Gölcük Natural Park, which has already been a subject of several papers, including i.a. records of two genera and 25 species new to Turkish fauna and among them five species of Encyrtidae (Hymenoptera: Chalcidoidea) described as new to science (Fent and Japoshvili 2012, Japoshvili 2011, Japoshvili and Anlas 2011, Japoshvili and Çelik 2010, Japoshvili and Ljubomirov 2011, Japoshvili and Toyganözü 2011, Japoshvili et al. 2009, 2010, 2011). Concerning Heteroptera, Fent and Japoshvili (2012) identified 66 species of true bugs from 13 families, the family Coreidae being represented by 9 species (7 in the tribe Pseudophloeini).

## Material and methods

**Figure 1.**
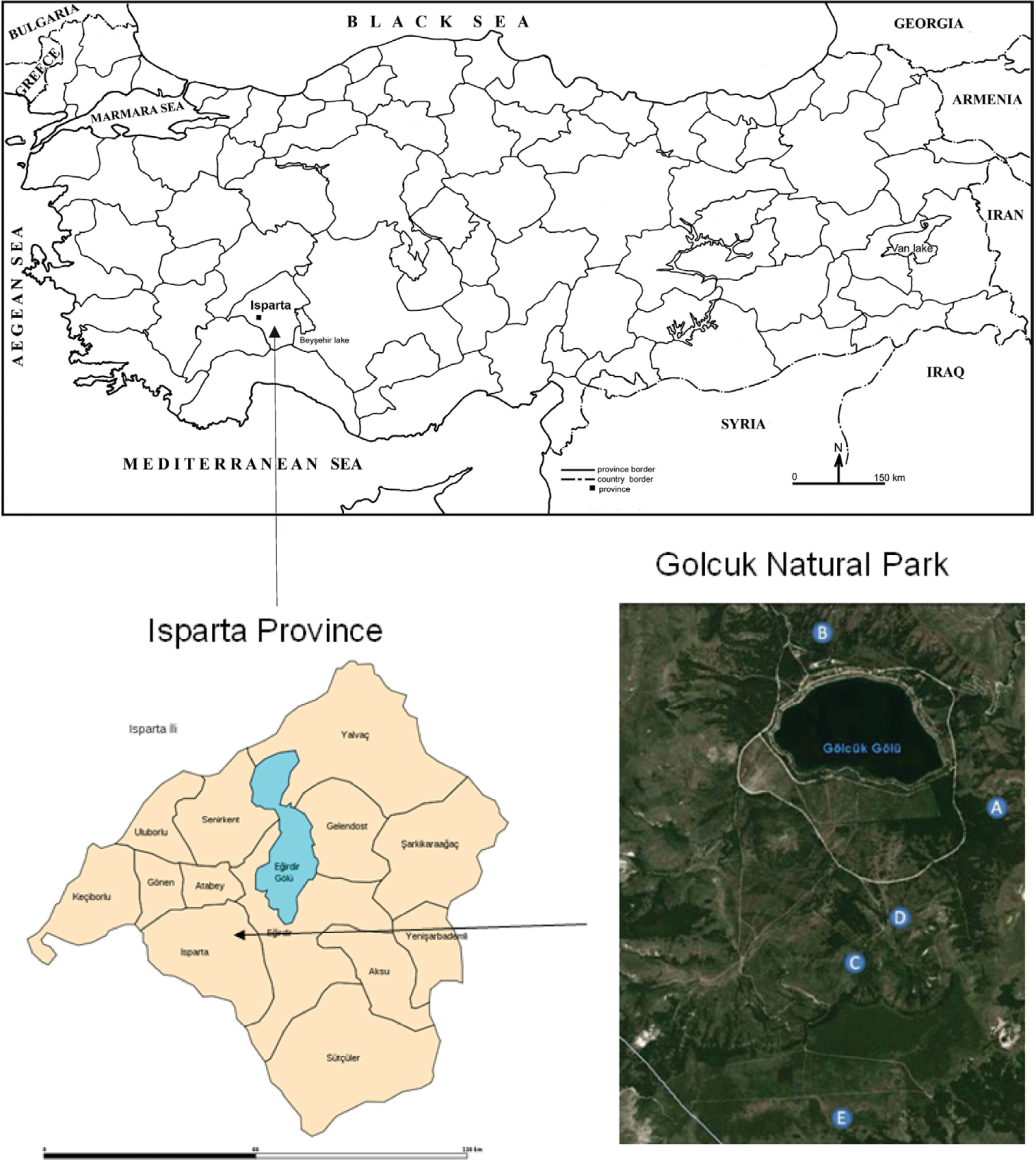
Location of the Gölcük Natural Park within Turkey and position of the collection sites of *Urartucoris ermolenkoi* P. V. Putshkov, 1979 within the Natural Park.

The Gölcük Natural Park (GNP) (Fig. 1) is situated in Isparta Province (Mediterranean Region of Turkey) in an arid region located 8 km southwest of the city Isparta. With its diverse vegetation and wildlife, geomorphological structure, and aesthetically pleasing landscape, GNP is one of the most important areas of the Lakes District in Turkey (Fig. 17). This area of 5,925 ha was proclaimed a natural park but its condition is deteriorating because it has no master plan and only minimal management (Gül et al. 2005). Area of the GNP has a rather complex geology, composed of alternating sedimentary (Akdağ limestone, conglomerates, flysch), magmatic (harzburgite, serpentinite), and volcanic rocks (trachy-andesites, tight tuffs, ash, and pumice tuff stones). Isparta Province itself is located at the border between the Irano-Anatolian and Mediterranean basin biodiversity hotspots, and this is reflected in the flora of the GNP as well: 22 (9.7 %) species endemic for Irano-Anatolian hotspot and 17 (7.5 %) endemic for Mediterranean basin hotspot are represented in this region (Fakir 1998, Fakir and Dutkuner 1999); 25 species (11 %) are endemic for Turkey (Fakir 1998). The studies performed around Gölcük Lake showed that 227 plant taxa from 136 genera within 47 families existed there, and among them red pine (*Pinus brutia* Ten.), black pine (*Pinus nigra* Arnold. ssp. *pallasiana* (Lamb.), oaks (*Quercus* spp.), cedar (*Cedrus libani* A. Rich.), pseudoacacia (*Robinia pseudoacacia* L.) and some other shrubs are characteristic for the Gölcük Natural Park (Fakir and Dutkuner 1999, Karatepe et al. 2005).

Dry-mounted specimens were measured under a stereomicroscope using an ocular micrometer. The following measurements were examined: body length (from apex of clypeus to apex of membrane), head length (from apex of clypeus to the anterior pronotal margin), head width (maximum width across eyes), interocular width (between inner margins of eyes), lengths of antennomeres (maximum lengths), pronotum length (medially in most exposed view), pronotum width (maximum width between humeral angles), scutellum length (medially from base to apex), scutellum width (maximum width at base), and abdomen width (maximum width).

All line drawings (using camera lucida) and the dissections of genitalia were made under a Leica MZ75 stereomicroscope. For the study of genitalia, a male specimen was softened in distilled water, and the pygophore was removed under stereomicroscope using a fine forceps, then put into concentrated KOH solution and heated until the solution started to boil. After maceration the pygophore was washed in distilled water and dissected under stereomicroscope. The dissected phallus is preserved in a plastic microvial with glycerol attached to the same pin as the specimen. The morphological terminology follows mostly Tsai et al. (2011).

## Results

### Urartucoris

P. V. Putshkov, 1979

http://species-id.net/wiki/Urartucoris

Urartucoris P. V. Putshkov, 1979: 62 (original description). Type species:*Urartucoris ermolenkoi* P. V. Putshkov, 1979, by original designation.
Urartucoris : Dolling (1986): 206–207 (taxonomic relationships, distribution), Dolling (2006): 55 (catalogue).

#### Redescription.

**Figures 2–5.**
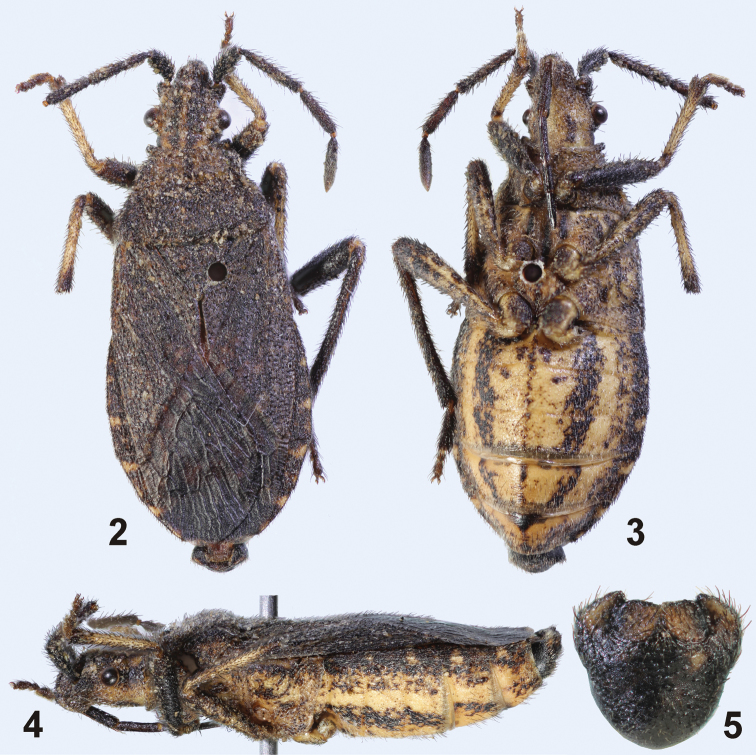
*Urartucoris ermolenkoi* P. V. Putshkov, 1979, male (10.2 mm). **2–4** habitus (**2** dorsal view **3** ventral view **4** lateral view) **5** intact pygophore in posterior view. (Photos P. Kment).

***Structure*.** *Head* porrect, robust, about as long as wide across eyes, strongly gibbose dorsally, anterior portion of head (anteriad of antenniferous tubercles) long (Fig. 2). Clypeus anteriorly surpassing mandibular plates. Antenniferous tubercles large, apically produced into long, inward-curved projection embracing base of antennal segment I. Compound eyes small, globular, protruding from head outline by most of their width (Fig. 2). Ocelli situated on small tubercles slightly posteriad of posterior margin of compound eyes, directed dorsolaterad; each ocellus closer to eye than to each other. Antennal segments ordered from longest to shortest: II > IV > III ≥ I. Antennal segment I robust, obovate, narrowing in basal one quarter of its length, slightly curved towards base, its apex surpassing apex of clypeus anteriorly; antennal segments II–IV much more slender, II and III almost cylindrical, slightly widening towards apex, IV spindle-shaped, with constricted base (Fig. 2). Bucculae short, covering approximately anterior half of labial segment I, surpassing labial segment I ventrally, ventral margin rounded, anteriorly reaching apex of clypeus (Fig. 4). Labial segments ordered from longest to shortest: I > II > IV > III; apex of segment I not reaching posterior margin of head, apex of segment II reaching anterior margin of procoxae, and apex of segment IV reaching anterior margin of mesocoxae (Figs 3, 4).

*Pronotum* trapezoid, anterior margin slightly concave, lateral and posterior margin nearly straight (Fig. 2). Pronotum highest at line connecting humeral angles, sloping anteriorly towards head (Fig. 4). Pronotal disk flat, slightly sloping towards lateral and posterior margin. Anterior margin of pronotum raised, forming sharp collar (most prominent laterally) (Figs 2, 3), constricted posteriorly by deep transverse groove continuing to propleura; anterolateral angles of pronotum such as in *Ceraleptus* not developed. Lateral margins of pronotum and humeral angles rounded, unarmed, not protruding (Fig. 2).

*Scutellum* triangular, slightly wider than long, flat, only anterolateral angles with small depressions, apex acutangulate (Fig. 2).

*Thoracic venter*. Mesosternum depressed between mesocoxae. Metasternum anteriorly convex, narrowing posteriad, metacoxae situated close to each other (Fig. 3). Metapleuron posterolaterally rounded, not protruding (Fig. 3). Ostiole of metathora- cic scent glands shifted somewhat laterad, situated between meso- and metacetabulum, laterally accompanied with a short peritreme; vestibular scar well visible; evaporatorium very small, narrowly surrounding vestibular scar, ostiole, and peritreme.

*Legs*. All femora oval in cross-section. Profemur widest in midlength, mesofemur approximately in its apical third (Fig. 3), both unarmed. Metafemur clavate, widest subapically (Fig. 3), its ventral surface with two parallel rows of more than ten spines and small denticles getting bigger from base to apex, two to four of the spines being large, the spines in rows being situated in nearly equal distances; surface between both rows flat, smooth. Tibiae somewhat flattened laterally, slightly widening from base to apex, unarmed. Tarsomeres ordered from longest to shortest: I > III > II, tarsomere I being slightly longer than II and III combined (Fig. 3).

*Wings*. Corium widest approximately at midlength, narrowing both anteriad and posteriad, costal margin of corium therefore slightly convex medially; posterolateral angle of corium acutangulate (Fig. 2). Membrane apically rounded, reaching apex of abdomen (♂; Fig. 2) or slightly shorter (♀). Hind wings developed.

*Abdomen* widest slightly behind its midlength (Fig. 3). Corium exposed, directed dorsolaterad, its outer margin smooth, posterolateral angles of laterotergites not protruding (Fig. 3), except for obtusangulate posterolateral angles of laterotergite VII in females. Abdominal venter regularly convex.

**Figures 6–9.**
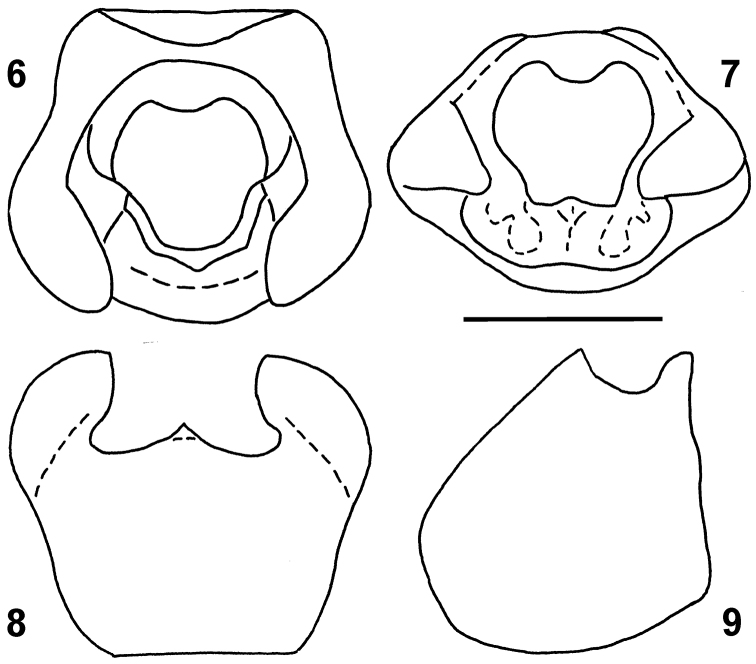
*Urartucoris ermolenkoi* P. V. Putshkov, 1979, dissected pygophore: **6** dorsal view **7** posterodorsal view **8** posterior view **9** lateral view. Scale bar: 1 mm.

**Figures 10–16.**
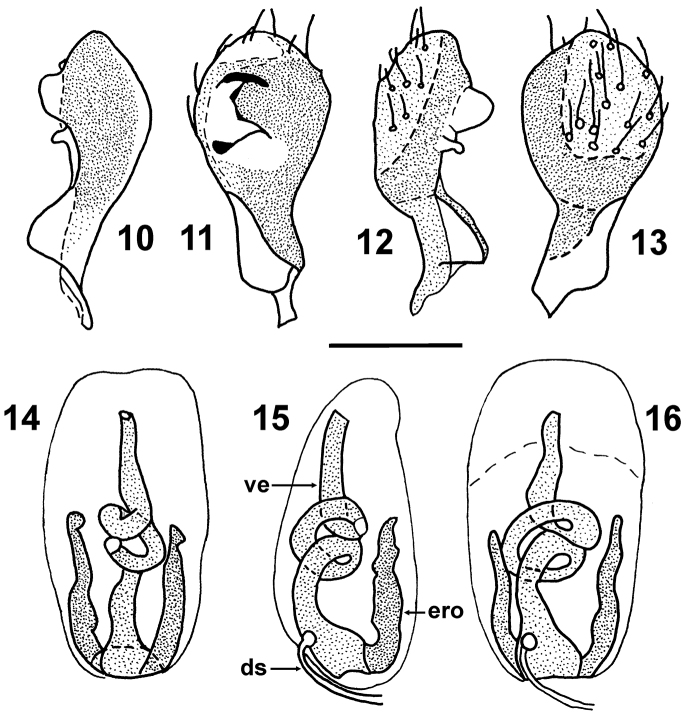
*Urartucoris ermolenkoi* P. V. Putshkov, 1979. **10–13** paramere (**10, 12** lateral views **11** anterior view **13** posterior view). **14–16** phallus (not inflated, articulatory apparatus lost) **14** dorsal view **15** lateral view **16** ventral view). Lettering: **ero** endophallic reservoir outgrowths, **ds** ductus seminis, **ve** vesica. Scale bar: 0.5 mm.

*Male genitalia*. Pygophore (Figs 5–9) black, lateral angles slightly brownish, insinuated anterolaterally, posterolateral angles distinctly produced, lobe-like, surrounding parameres laterally; infolding of ventral rim large, with a pair of depressions harbouring basal portion of parameres (Fig. 7). Paramere sockets not visible in dorsal view, covered by posterolateral angles of pygophore (Figs 6–7). Paramere (Figs 10–13) clavate in posterior (outer) and anterior (inner) view, slightly S-shaped in lateral view; posterior surface (Fig. 13) of head of paramere flattened, pale brown, bearing sparse and stout setae arising from large punctures; rest of paramere body blackish; inner surface (Fig. 11) produced into two ridges holding acute angle, distal ridge higher, apically rounded, proximal one lower and angulate; surface between the ridges and between proximal ridge and base of the paramere concave, rest of anterior surface convex. Phallus (Figs 14–16) with sclerotized vesica with two coils and a single pair of long endophallic reservoir outgrowths.

#### Differential diagnosis.

*Urartucoris* differs from all Palaearctic Pseudophloeini in very long antennal segment II, sharp and well delimited pronotal collar, and presence of two nearly identical rows of denticles and spines on metafemora, the spines in the rows being situated in nearly equal distances. It resembles the genus *Ceraleptus* (especially *Ceraleptus gracilicornis* (Herrich-Schaeffer, 1835)) in the close position of the metacoxae but it differs from it, besides the above mentioned generic characters, in the robust antennae and the body being covered by stiff spinules (Putshkov 1979). See also the Key below. Also Dolling (1986) suggested close relationship between *Urartucoris*, *Ceraleptus*, and *Microtelocerus*, but without listing a single shared character.

#### Etymology.

Originally, etymology of the name was not specified. The name consists of the name Urartu, which was an ancient Armenian kingdom (ca. 860–585 B.C.) spread out between Asia Minor, Caucasus and Mesopotamia, with center around the Van Lake (today in eastern Turkey), and the ending -*coris*, used for true bug. The name is masculine.

### Urartucoris ermolenkoi

P. V. Putshkov, 1979

http://species-id.net/wiki/Urartucoris_ermolenkoi

Figs 2 – 16

Urartucoris ermolenkoi P. V. Putshkov, 1979: 63–64. Type material: Holotype: ♀, Azerbaijan: ‘Nakhichevanskaya ASSR: 6 km N of Bilav village, 1700 m, 22 V 1966 (V. M. Ermolenko)’ (coll. Institute of Zoology, Ukrainian Academy of Sciences, Kiev); paratype: 1 ♀, Azerbaijan, Nakhchivan: ‘0.5 km E of Ak-Dar village, 2000 m, 19 VII 1977 (P. V. Puchkov)’ (coll. Zoological Institute, Russian Academy of Sciences, St. Petersburg).
Urartucoris ermolenkoi : Dolling (2005): 55 (catalogue), Putshkov and Putshkov (2012): 95 (type depository).

#### Material examined.

**TURKEY: Isparta province:** Gölcük (site A), 17.iv.2008, 1 ♂, M. Kaya lgt.; (sites A,B), 17.iv.2008, 3 ♂♂, M. Kaya lgt.; (site D), 24.iv.2008, 1 ♂ 1 ♀, G. Japoshvili lgt.; (site C), 15.v.2008, 1 ♀, M. Kaya lgt.; (site E), 10.vii.2008, 1 ♂ 1 ♀, G. Japoshvili lgt.; (site B), 24.vii.2008, 1 ♀, G. Japoshvili lgt.; (site E), 11.ix.2008, 1 ♂ 1 ♀, G. Japoshvili lgt. (coll. Trakya University, Edirne, Turkey, except 1 ♂ 1 ♀ in coll. National Museum, Praha, Czech Republic).

Location of the collecting sites: A – 37°43'33.81"N, 30°30'26.22"E, 1472 m alt.; B – 37°44'13.12"N, 30°29'22.95"E, 1420 m alt.; C – 37°42'49.02"N, 30°29'48.93"E, 1485 m alt.; D – 37°43'03.00"N, 30°29'56.90"E, 1443 m alt.; E – 37°42'09.05"N, 30°29'43.97"E, 1621 m alt.

#### Redescription.

***Colouration*** (Figs 2–5).Body dorsally dark brown, except three ochraceous stripes dorsally on head, one in midline, running from base of head towards base of clypeus, and two lateral ones, running from base of head along inner margin of eye towards base of antenniferous tubercle; elongate ivory spot on apex of scutellum; and rather irregular whitish spots posterolaterally on laterotergites. Membrane brownish, with small round pale spots; veins dark brown. Antennae and labium black. Head ventrally brown. Thorax ventrally dark brown, pleura to various extent covered with smaller to larger, irregular, sometimes confluent ochraceous spots, especially on metapleuron. Profemora, metatibiae and metatarsi blackish brown. Meso- and metafemora blackish brown with irregular ochraceous spots, especially on dorsal surface. Pro- and mesotibiae ochraceous, basally and apically infuscated with dark brown, pro- and mesotarsi dark brown. Abdomen ventrally pale with nearly continuous wide blackish stripes laterally and at mid-distance between lateral margin and midline, sternites III–VII with narrow, black, nearly continuous to interrupted stripe along midline.

*Measurements*. Males (mm; n = 8): Body length 9.4–10.3; head: length 1.7–1.8, width across eyes 1.7–1.8, interocular width 1.0–1.3; pronotum: length 1.9–2.1, width across pronotal collar 1.6–1.8, width across humeral angles 2.9–3.3; scutellum: length 1.1–1.3, width 1.4–1.6; abdomen: maximum width (slightly behind its midlength) 4.0–4.2; length of antennal segments: I – 0.9–1.1, II – 1.2–1.5, III – 0.9–1.1, IV – 1.0–1.2; profemur: length 1.8–2.2; protibia: length 1.7–2.0; mesofemur: length 2.1–2.5; mesotibia: length 2.0–2.3; metafemur: length 2.7–3.6; metatibia: length 3.0–3.5.

Females (mm; n = 4): Body length 10.5–11.0; head: length 1.7–1.9, width across eyes 1.8–2.0, interocular width 1.2–1.25; pronotum: length 2.0–2.1, width across pronotal collar 1.7–1.9, width across humeral angles 3.1–3.4; scutellum: length 1.2–1.4, width 1.5–1.7; abdomen: maximum width (slightly behind its midlength) 4.2–4.6; length of antennal segments: I – 0.9–1.1, II – 1.3–1.4, III – 1.1 (n = 1), IV – 1.2 (n = 1); profemur: length 2.0–2.3; protibia: length 1.9–2.2; mesofemur: length 2.3–2.6; mesotibia: length 2.1–2.3; metafemur: length 3.1–3.8; metatibia: length 3.0–3.4.

***Pilosity and vestiture*.** Body dorsum (except membrane), antennae and legs covered with long, stiff, semi-erect to erect, brown to black spinules, arising from apices of small tubercles (best visible in lateral view); tubercles largest on vertex and anterior portion of pronotum, those on posterior portion of pronotum, scutellum, and coriaceous part of hemelytra smaller. Spinules on tibiae nearly as long as half of diameter of tibia, those on femora nearly as long as the large spinules on pronotum. Body venter with double pilosity: Long, stiff, semi-erect to erect, dark spinules as those on body dorsum, but distinctly sparser, arising from smaller tubercles, short on pleura and ventral surface of head. Besides the dark spinules, body venter covered with intermingled, sparse, adpressed, whitish setae, slightly shorter than the spinules. Antennal segment IV covered with very short and fine adpressed pubescence among the sparse, long black spinulae. Besides the tubercles body covered with irregularly scattered, deep and dark punctures, largest on clavus.

#### Variability.

The male resembles the female in most of the characters except for slightly smaller body (9.4–10.3 mm) than in females (10.5–11.2 mm), membrane reaching apex of abdomen (slightly shorter in females) and shape of last abdominal segments. We found also some differences in colouration, but this may represent rather intraspecific variability than sexual dimorphism: Peritreme yellowish, only slightly infuscated on its lateral edge (♂); peritreme black (♀). Abdominal sternites III–VII with narrow, black, nearly continuous stripe along midline (♂); sternite III medially with large blackish spot, the black longitudinal stripe in ventral midline being interrupted, ventrites IV–VI medially with only smaller black spots posteriorly (♀). The extent of ochraceous colouration on thoracic pleura is certainly variable among specimens.

The Turkish specimens fit well the original description except for a few details. The mesofemora of the Turkish specimens are unarmed, while Putshkov (1979) mentioned mesofemora with two small spines. There are also slight differences in colouration. According to Putshkov (1979), the Nakhchivan specimens differed, e.g., in antennae dark brown with antennal segment I black; anterior portion of pronotum paler than its posterior portion, darkened near lateral margins and along midline; meso- and metafemora pale, apically darkened, especially dorsally; and abdomen ventrally pale with isolated dark spots, forming two interrupted stripes in lateral midlines (halfway between connexivum and ventral midline of abdomen). The Turkish females are either slightly smaller or approximately as large as the Nakhchivan specimens.

#### Etymology.

Originally, etymology of the name was not specified. Most probably, the species was dedicated to Valeriy Mikhaylovich Ermolenko (1920–2006), an Ukrainian expert in Hymenoptera: Symphyta and collector of the holotype.

#### Phenology.

Adults were collected from mid April to end of July and in mid September (Putshkov 1979, this paper).

#### Habitat.

All the specimens of *Urartucoris ermolenkoi* (all adults) were collected between April and September 2008 using pitfall traps; collecting by other methods (yellow traps) yielded no specimens of this apparently epigeic species. The species was collected at five different semi-natural collecting sites in higher altitudes (1420–1621 m a.s.l.), ranging from sparse forest to mountain grassland (Figs 18–20).

- **A** Xerophilic natural plants with a reforested area with pine trees (*Pinus* sp.) and cedars (*Cedrus* sp.) planted between 1959–1969 (Şahbudak & Cengiz 2007); about 4.8 % of the plants that were recorded from this site were endemic to Turkish flora. Altitude 1472 m (Fig. 18).
- **B** Main entrance to the GNP, this is an area close to the lake, with areas reforested with *Robinia pseudoacacia* planted between 1960–1965. Some natural plants like *Crataegus orientalis*, *Cotoneaster nummularia*, *Pistacia terebinthus* and other are also represented in this site which has high human activity (picnic area). Altitude 1420 m.
- **C** Mesophilous area with plantation of 50–60 years old *Populus* spp. trees, accompanied by *Crataegus orientalis*, *Cotoneaster nummularia*, *Pistacia terebinthus*, *Rosa canina*, *Pyrus*, *Juglans* and *Malus* spp. Altitude 1485 m.
- **D** Dry xerophilic sandy place with *Robinia pseudoacacia* plantations and natural shrubland with different dominant *Astragalus* spp., many of them endemic. Altitude 1443 m (Fig. 19).
- **E** Highland site, reforested in 1989 with *Cedrus* sp. and *Robinia pseudoacacia*. Altitude 1621 m (Fig. 20).

**Figures 17–18.**
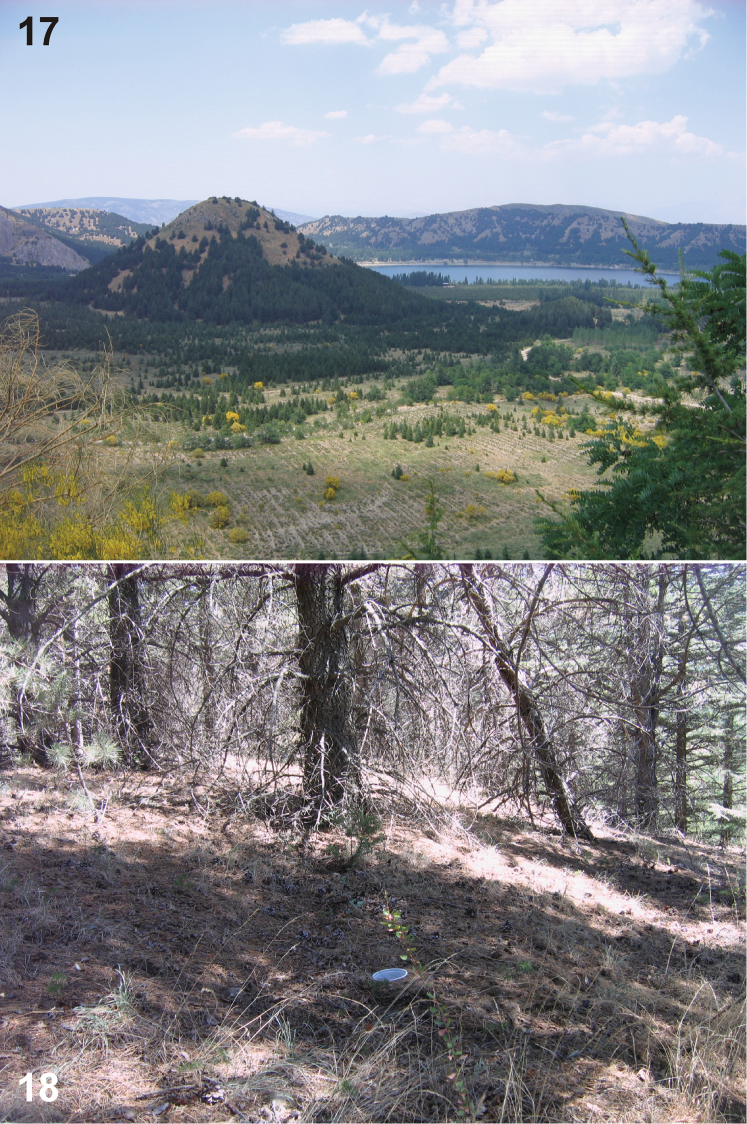
Habitats of *Urartucoris ermolenkoi* P. V. Putshkov, 1979. **17** Landscape of the Gölcük NP **18** collecting site A. (Photos G. Japoshvili).

**Figures 19–20.**
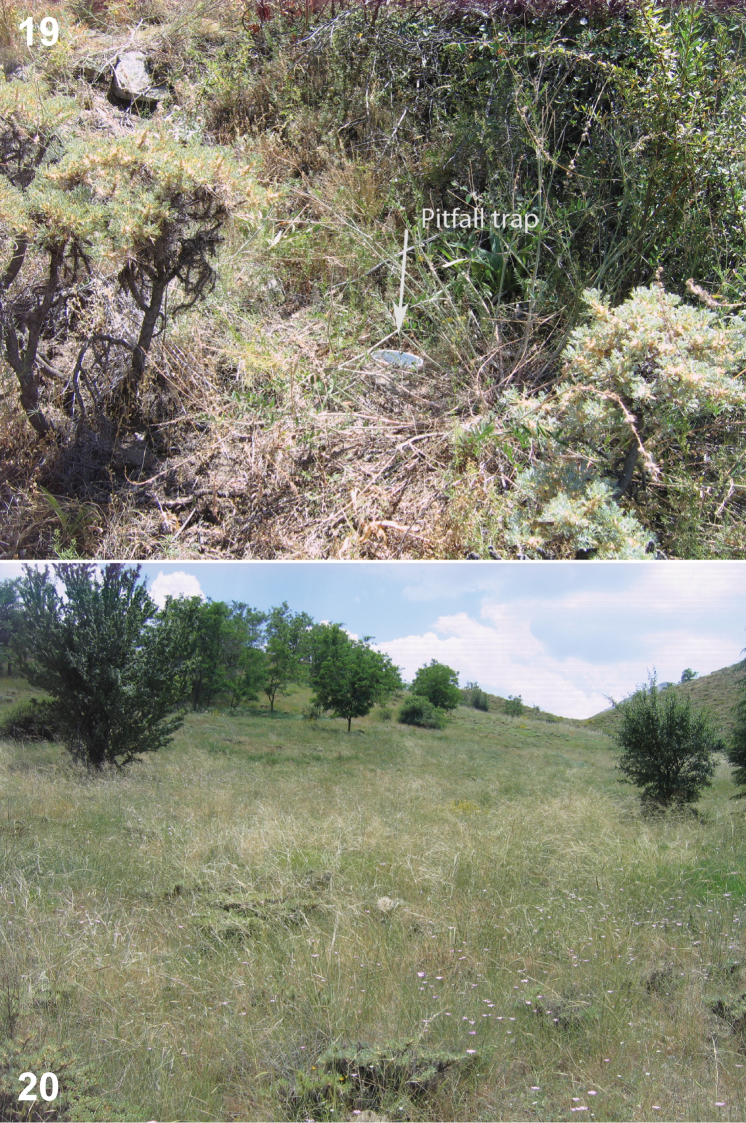
Microhabitats of *Urartucoris ermolenkoi* P. V. Putshkov, 1979, showing also placement of the pitfall trap **19** collecting site D **20** collecting site E. (Photos G. Japoshvili).

#### Distribution.

Asian Turkey (Isparta province) (this paper, see Fig. 1), Azerbaijan: Nakhchivan (Putshkov 1979).

### Revised key to the genera of the West Palaearctic Pseudophloeini

**Figures 21–23.**
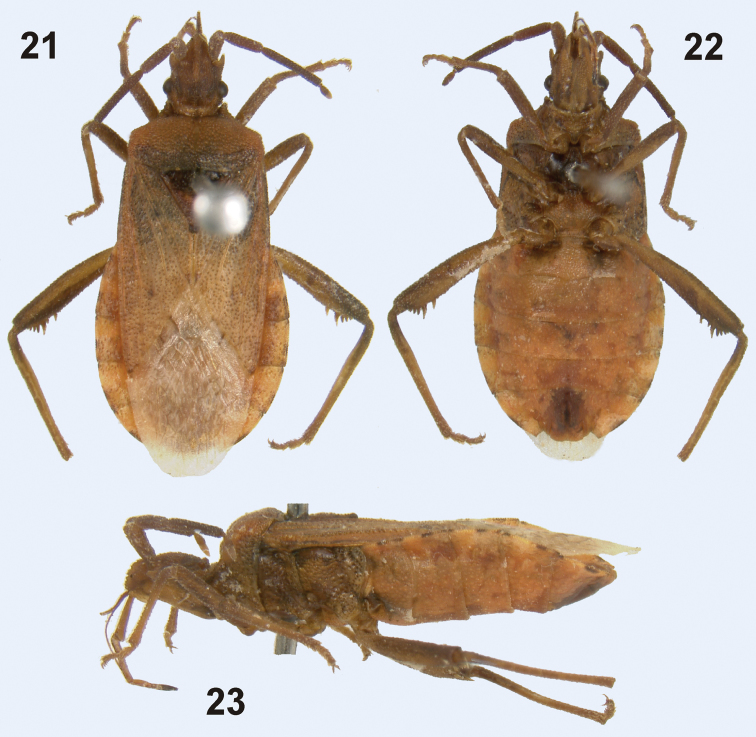
*Microtelocerus linnavuorii* Dolling, 1979, habitus of male holotype (**21** dorsal view **22** ventral view **23** lateral view). (Photos M. Wilson).

The following key is based on translation of Moulet (1995). The characters of *Microtelocerus* are based on examination of the male holotype of *Microtelocerus linnavuorii* deposited in the National Museum and Gallery of Wales, Cardiff (type labels: ‘Holo- / type [p, white label with red margin] // HEBREW UNIVERSITY OF JERUSALEM, ISRAEL / Department of Entomology [p] + m [hw] / LOC: [p] Gabal Sarbal 1300 [hw] / DATE: [p] 8.8.68 [hw] / COL: [p] Broza & Toren [hw, white label] // HOLOTYPE / Microtelocerus / Linnavuorii [hw] / det. [p] n. sp. M.S. [hw] / W.R. Dolling 197[p]2[hw, white label]’) (see Figs 21–23), supplemented by information from Reuter (1900), Dolling (1979) and the habitus illustration by Kiritshenko (1952: 169, fig. 18). The key allows identification of all Pseudophloeini genera occurring in the western half of the Palaearctic Region (up to Central Asia), except Yemen where two additional genera of Afrotropical origin occur – *Mevanidea* Reuter, 1882 and *Risbecocoris* Izzard, 1949 (Dolling 2006).

**Table:** 

1 (8)	Antennal segment III more than 2 times longer than segment II	2
2 (7)	Antennal segment III much (3–4 times) longer than segment II	3
3 (4)	Posterior margin of pronotum with a spine at each side of scutellum. Head and pronotum with long setae	*Strobilotoma* Fieber, 1860 (1 species)
4 (3)	Posterior margin of pronotum without spines. Head and pronotum with short setae	5
5 (6)	Metafemora tuberculate, generally without or at most with an inconspicuous spine apically. Lateral margins of pronotum concave medially	*Arenocoris* Hahn, 1834(4 species)
6 (5)	Metafemora smooth with a strong apical spine. Lateral margins of pronotum straight	*Bathysolen* Fieber, 1860 (2 species)
7 (2)	Antennal segment III 2.5 times longer than segment II	*Ulmicola* Kirkaldy, 1909 (1 species)
8 (1)	Antennal segments II and III subequal, rarely antennal segment III longer (no more than 2 times) or shorter than segment II	9
9 (22)	Antennal segments II and III subequal, rarely antennal segment III longer (no more than 2 times). Metafemora without two parallel rows of spines ventrally	10
10 (11)	Posterior margin of pronotum conspicuously denticulate	*Coriomeris* Westwood, 1842 (12 species)
11 (10)	Posterior margin of pronotum unarmed.	12
12 (17)	Antenniferous tubercles terminating in spine directed more or less distinctly forward	13
13 (14)	Antennal segment IV distinctly longer and thicker than III. Pro- and meso-femora dentate, with one big and few small spines subapically. Head dorsally, pronotum and scutellum covered with long and dense pubescence	*Loxocnemis* Fieber, 1860 (1 species)
14 (13)	Antennal segment IV distinctly shorter and only slightly thicker than antennal segment III. Pro- and mesofemora unarmed or granulate, mesofemora with only one small spine or without a spine subapically. Head dorsally, pronotum and scutellum at most with short pubescence	15
15 (14)	Antennal segment IV less shorter (0.75–0.85 times) and thicker than antennal segment III. Profemora and mesofemora with only one small spine subapically or unarmed	*Anoplocerus* Kiritshenko, 1926(3 species)
16 (15)	Antennal segment IV much shorter (0.42 times) and slender than antennal segment III. Pro- and mesofemora unarmed	*Microtelocerus* Reuter, 1900(1 species; Figs 21–23)
17 (12)	Antenniferous tubercles obtuse or, if terminating in spine, this is curved inwards, hook-shaped.	18
18 (19)	Antennal segment I with conspicuous spine-like tubercles laterally, each with an apical seta. Humeral angles of pronotum armed with a distinct tooth. Mandibular plates reaching anterior margin of clypeus, well developed	*Bothrostethus* Fieber, 1860 (1 species)
19 (18)	Antennal segment I without such tubercles. Humeral angles of pronotum unarmed. Mandibular plates not conspicuously developed	20
20 (21)	Metafemora with group of spines apically, 2–3 of the spines larger than remaining ones	*Ceraleptus* A. Costa, 1847 (5 species)
21 (20)	Metafemora with a single spine apically	*Nemocoris* R. F. Sahlberg, 1848 (1 species)
22 (9)	Antennal segment II distinctly longer than segment III. Metafemora bearing two parallel rows of spines on their ventral surface, between which the tibia could rest.	*Urartucoris* P. V. Putshkov, 1979 (1 species; Figs 2–4)

## Supplementary Material

XML Treatment for Urartucoris

XML Treatment for Urartucoris ermolenkoi

## Appendix

Translation of the original descriptions published in Russian (Putshkov 1979):

***Urartucoris* P. Putshkov, gen. n.**

**Type species.** *Urartucoris ermolenkoi* P. Putshkov, sp. n.

Entire body completely scattered over with hard black spinules originating from small tubercles, which are less developed on ventral surface of the body. Width of head across eyes nearly equal to head length. Antennae robust, covered with hard spinules, and so are the legs. Antennal segment II distinctly longer than segment III. Pronotum slightly sloping anteriad, its anterior margin forming a collar, divided from lateral margins by deep furrow, which is best visible in dorsal view. All margins of pronotum without setae, its lateral angles rounded, without spines or processes. Metacoxae convergent. Metafemora bearing two parallel rows of spines on their posterior surface, between which the tibia can rest.

The new genus belong to the subfamily Pseudophloeinae, differing from all its Pa-laearctic representatives in very long antennal segment II, wide pronotal collar, and two nearly identical rows of spines on metafemora, the spines in the rows stand in nearly equal distances. It resembles the genus *Ceraleptus* (especially *Ceraleptus gracilicornis* H.-S.) in the degree of convergence of metacoxae but it differs, apart from the above mentioned generic characters, in robust antennae and body covered with hard spinules.

***Urartucoris ermolenkoi* P. Putshkov, sp. n.** (see figure)

Body dorsally blackish-brown with dense black punctures, ventrally pale brown with black spots. Body length 11–11.2 mm, body width (at level of last quarter of body) 4.5–4.8 mm. Head length 1.95–2.1 mm, head width including eyes 1.9 mm, between eyes 1.5 mm. Total length of antennae 5–5.5 mm, ratio of antennal segments 22–28: 30–33: 22–25: 24–25. Lateral processes of antenniferous tubercles bent around antennal bases in form of annulus, in dorsal view it seems that antennae originate at bottom of a bowl. Tubercles on head big, bearing setae 2–3 times longer than height of tubercles. Antennae dark brown with antennal segment I black. Antennal segment I obovate, elongate, slightly curved towards base, apically 1.3–1.4 times wider than segment II. Segments II and III gradually widening (from 0.15 to 0.24 mm) towards apex, as wide as segment IV. Segment II 1.35 as long as segment III. Setae covering antennae nearly as long as width of segments II and III. Rostrum reaching mesocoxae.

Length of pronotum 2.1 mm, width across humeral angles 3.4–3.65 mm, across pronotal collar 1.9 mm, equally wide as head across eyes. Anterior portion of pronotum bearing large setiferous tubercles, the same as on the head. Anterior portion of pronotum paler than its posterior portion, darkened near lateral margins and along midline. Tubercles on the posterior portion of the pronotal disk lower, setae shorter, punctures more dense and darker. Sculpture of scutellum and the coriaceous part of hemelytra the same as on posterior surface of pronotum, except the punctures on clavus being larger.

Metacoxae convergent, as far as 2/3 of width of tibia or 1/4 of width of coxal cavity. Spines in both rows on metafemora nearly the same, regularly shortening towards base of femur. Each row including more than ten spines, two to four being large. Mesofemora with two small spines or none. Profemora completely dark, meso- and metafemora pale, apically darkened, especially dorsally. Metatibiae darker than pro- and mesotibiae. Tarsi dark, length ratio of metatarsal segments 15: 4: 7. Legs covered with black semierect setae, setae on tibiae nearly as long as half of the tibia width, those on femora nearly as long as the large spines.

Abdomen ventrally pale with isolated dark spots, forming two interrupted stripes in lateral midlines (half the distance between connexivum and ventral midline of abdomen). Connexivum dark with pale spots.

Material. 1 ♀ (holotype), Nakhichevanskaya ASSR: 6 km north of village Bilav, 1700 m, 22 V 1966 (V. M. Ermolenko); 1 ♀, 0.5 km east of village Ak-Dar, 2000 m, 19 VII 1977 (P. V. Putshkov). Holotype deposited in Institute of Zoology, AN USSR, and paratype in Zoological Institute AN SSSR in Leningrad.
